# Open Versus Laparoscopic Pyloromyotomy for Hypertrophic Pyloric Stenosis: A Systematic Review and Meta-Analysis Focusing on Major Complications

**DOI:** 10.1007/s00464-012-2174-y

**Published:** 2012-02-21

**Authors:** M. W. N. Oomen, L. T. Hoekstra, R. Bakx, D. T. Ubbink, H. A. Heij

**Affiliations:** 1 Department of Surgery (Surgical Laboratory), Academic Medical Center, Meibergdreef 9, 1105 AZ Amsterdam, The Netherlands; 2 Departments of Quality Assurance and Process Innovation and Surgery, Academic Medical Center, Amsterdam, The Netherlands; 3 Department of Pediatric Surgery, Academic Medical Center, Meibergdreef 9, 1105 AZ Amsterdam, The Netherlands

**Keywords:** Abdominal, Pediatric, Endoscopy, Complications, Pyloric, Review

## Abstract

**Background:**

There is an ongoing debate about whether laparoscopic pyloromyotomy (LP) or open pyloromyotomy (OP) is the best option for treating hypertrophic pyloric stenosis (HPS). The aim of this study was to compare the results of both surgical strategies by means of a systematic review and meta-analysis of the available literature.

**Methods:**

A systematic search for randomized clinical trials (RCTs) comparing OP and LP was conducted. Studies were reviewed independently for quality, inclusion and exclusion criteria, and outcomes. Primary outcome was major postoperative complications (i.e., incomplete pyloromyotomy, perforation, and need for reoperation). Secondary outcomes were time to full feed, postoperative hospital stay, and any other postoperative complications.

**Results:**

Four RCTs with a total of 502 patients (OP 255, LP 247) fulfilled the inclusion criteria and were analyzed in this review. These trials showed an absolute incidence of major postoperative complications of 4.9% in the LP group. Meta-analysis showed that LP did not lead to significantly more major postoperative complications (ARR 3%, 95% CI −3 to 8%) than OP. The mean difference in time to full feed was significant (2.27 h, 95% CI −4.26 to −0.29 h) and the mean difference in postoperative hospital stay tended to be shorter (2.41 h, 95% CI −6.10 to 1.28 h), both in favor of LP.

**Conclusion:**

So far, the major postoperative complication rate after LP for HPS is not substantially higher than after OP. Because time to full feed and postoperative hospital stay are at best a few hours shorter after LP than after OP, the laparoscopic technique might be acknowledged as the standard of care if the major postoperative complication rate is low. Hence, this laparoscopic procedure should preferably be performed in centers with pediatric surgeons with expertise in this procedure.

Hypertrophic pyloric stenosis (HPS) is a common problem that is often seen in daily care in the pediatric surgical unit. The incidence of HPS is approximately 1–3 per 1,000 live births [1]. HPS is seen more often in males, with a male-to-female ratio of 4:1 [2]. The surgical treatment of choice in the last century has been the longitudinal splitting of the seromuscular layer of the pylorus without suturing, which is defined as “pyloromyotomy.” The constriction is relieved and allows normal passage of stomach contents into the duodenum. The operation traditionally has been performed through a classical right-upper-quadrant (RUQ) transverse incision. This operation is effective at providing excellent exposure of the pylorus but results in an abdominal scar that grows with the patient and becomes quite significant with time.

Several other approaches have been introduced, such as that described by Tan and Bianchi [3] in which the pyloromyotomy is performed through a supraumbilical skin fold incision. This technique achieves an excellent cosmetic outcome with an apparently unscarred abdomen. Alain et al. [4] introduced the laparoscopic approach in 1991. Both surgical modalities have gained wide acceptance in the western world. The potential advantages of the laparoscopic pyloromyotomy (LP) are shorter hospital stay, improved cosmesis, shorter postoperative recovery, lower complication rates, and less postoperative pain [4–13]. These studies had different primary outcomes and subsequently reported advantages in favor of LP. None had complications as a primary outcome.

However, recently a review was published in which a difference in time to full feed of 12 h (3 h if only randomized clinical trials [RCTs] were encountered) and an earlier hospital discharge of 6 h (4 h if only RCTs were included) was found [12]. Both do not seem to offer convincing clinical relevance to promote LP apart from the cosmetic advantage. This review included complications, but the reduced complication rates in the LP group were due to mainly wound complications. In our opinion, a valid argument in favor of LP could be a reduction in major postoperative complications. The question arises if LP is a better operation technique for HPS in terms of postoperative complications and is therefore superior to the open approach.

Therefore, the aim of this study was to compare the results of LP and open pyloromyotomy (OP) by means of a systematic review of the available randomized trials while focusing on major complications (i.e., incomplete pyloromyotomy, perforation, and need for re-operation).

## Materials and Methods

### Search Strategy

A systematic search for RCTs that compared open and LP was conducted. Retrieval of studies was performed through a systematic search of the databases PubMed, Ovid (Ovid Technologies, New York, NY) and Cochrane (Cochrane database of systematic reviews). Keywords and medical subject heading (MeSH) terms used were “pyloric stenosis,” “pyloromyotomy,” “comparative studies,” “open,” “laparoscopic,” and “postoperative complications.” The full texts of the studies were read to determine whether the studies met the inclusion criteria. The reference lists of all articles that dealt with the topic of interest were scanned to check for additional publications. Disagreements about the inclusion of studies were resolved by group discussion (MWNO, RB, LTH). There were no language restrictions. No unpublished data were encountered.

### Study Selection

Potentially eligible studies were reviewed independently by two authors (MWNO, RB) for inclusion and exclusion criteria. Studies were included in the review if they were RCTs that compared the results of LP and OP in children with HPS with admission time after pyloromyotomy and postoperative complication rate as outcomes. The primary outcome was major postoperative complications (i.e., incomplete pyloromyotomy, perforation, and need for reoperation). Secondary outcomes were time to full feed, postoperative hospital stay, and any other postoperative complications.

### Data Collection

From the included studies, data on setting, methodological quality [according to the Cochrane handbook for systematic reviews of interventions (http://dcc.cochrane.org/sites/dcc.cochrane.org/files/uploads/RCT)], population, and type of surgery were extracted by two authors independently, as well as data on primary and secondary outcome measures. The reporting checklist proposed by the Consolidated Standards of Reporting Trials (CONSORT) group [14, 15] was used as a guideline when performing this review.

### Statistical Methods

Review Manager (RevMan) software ver. 5.0 (The Nordic Cochrane Center, The Cochrane Collaboration, Copenhagen, 2008) was used for data entry and statistical analysis. Continuous data are expressed as mean differences, with standard deviations (SD) or medians and interquartile ranges (IQR) where appropriate. Results for comparisons of dichotomous outcomes (e.g., major postoperative complications) are expressed as risk differences [or absolute risk reduction, ARR) with 95% confidence intervals (CI)]. A meta-analysis was planned if the included studies were clinically homogeneous. Statistical heterogeneity in the meta-analysis was assessed with the χ^2^ test and the *I* ^2^ index. If *I* ^2^ was above 30%, a random-effects approach instead of a fixed-effect analysis would be undertaken. If *I* ^2^ was over 60%, we would refrain from meta-analysis.

## Results

**Fig. 1:**
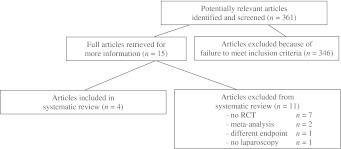
Number of articles identified and screened in the systematic review

The initial search yielded 361 potentially relevant articles, of which 346 articles were excluded because of failure to meet the inclusion criteria (Fig. 1). Fifteen full papers were retrieved for more information of which 11 studies were excluded from the systematic review. These excluded articles were not randomized controlled trials [5, 11, 16–18], consisted only of a meta-analysis [19], used different end points [20] or different treatment strategies [21] and a RCT in which no laparoscopy was performed. Four RCTs with a total of 502 patients (OP 255, LP 247) fulfilled the inclusion criteria and were analyzed in this review [6–8, 13]. Study details and the quality check of all RCTs are given in Tables 1, 2, respectively.

**Table 1:** Study details of all RCTs

Reference	*n*(OP, LP)	Center type (*n*)	Surgical technique
Hall et al. [7]	180 (93, 87)	Multicenter (6)	OP: Tan and Bianchi [3]
			LP: Najmaldin and Tan [22]
Leclair et al. [8]	102 (52, 50)	Single center	OP: Longitudinal seromuscular incision
			LP: Umbilical incision, avascular plane
St. Peter et al. [13]	200 (100, 100)	Single center	OP: According to surgeon’s personal technique
			LP: Umbilical stab incision technique
Greason et al. [6]	20 (10, 10)	Single center	OP: Umbilical fold incision
			LP: Modified version of Najmaldin and Tan [22]
			Superior umbilical fold region

**Table 2:** Quality check of all RCTs

Reference	Randomization	Blinded	Allocation concealment	Follow-up (range)
Hall et al. [7]	Randomly assigned	Double-blind	Facsimile communication with leading center or online via website^b^	39 days^a^ (32–51, 12–179) (*n* = 151)
Leclair et al. [8]	Sealed numbered envelopes	Double-blind		4–9 weeks (*n* = 102)
St. Peter et al. [13]	Non-stratified sequence in blocks of ten	No blinding	Operation discussed with family	
Greason et al. [6]	Sealed numbered envelopes			

^a^Median results after discharge allocation criteria according to the Cochrane handbook for systematic reviews of interventions

^b^Patients were randomized; the surgical procedure was blinded to parents and caregivers; patient characteristics were comparable; 84% of the patients attended a follow-up appointment; analysis was performed according to the assigned group; patients were treated equally in both groups

^c^An individual unit of randomization in a non-stratified sequence was used; the operation was blinded to patients; health-care professionals were aware of the treatment assigned; there were no differences between the groups at the beginning of the study; the follow-up was complete in both groups; all patients were analyzed according to the group in which they were allocated; there were no differences in treatment, besides the procedure

### Postoperative Complications

#### Major Postoperative Complications

**Fig. 2:**
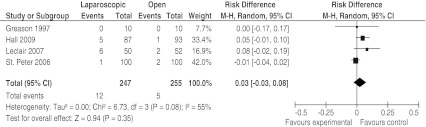
Forest plot of major postoperative complications in infants treated with OP and LP

All studies included in this review reported major complications, with a total of 12 (4.9%) major complications in children who underwent LP and 5 (2.0%) in the OP group [6–8, 13]. Using a random-effects approach, we found no significant difference between LP and OP (ARR 3%, 95% CI −3–8%). The forest plot comparing major postoperative complications is shown in Fig. 2.

#### All Postoperative Complications

**Fig. 3:**
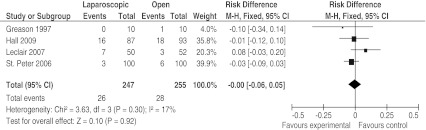
Forest plot of all postoperative complications

All four studies described postoperative complications, with only one complication in the OP group in the study by Greason et al. [6], which did not require treatment [6–8, 13]. In summary, a nonsignificant difference was found of 26 (10.5%) complications in the LP group versus 28 (11.0%) complications in the OP group. A forest plot is shown in Fig. 3.

### Time to Full Feed

**Fig. 4:**
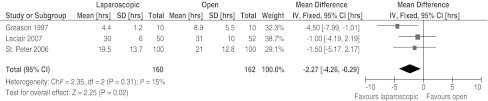
Forest plot of time to full feed in hours

Three RCTs reported on the results of the mean time after surgery to return to full feedings [6, 8, 13]. Two studies [8, 13] showed no difference in time to full feed between LP and OP and one study [6] did report a difference. The mean time to full feeding in this article was less in the LP group (4.4 h) than in the OP group (8.9 h). The data of Hall et al. [7] were medians and thus not suitable for the random-effects model. In conclusion, the mean difference in time to full feed was 2.27 h in our review, in favor of LP (Fig. 4). This difference was statistically significant (95% CI 0.29–4.26 h).

### Postoperative Hospital Stay

**Fig. 5:**
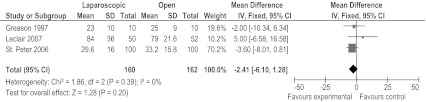
Forest plot of length of postoperative stay in hours

There were no differences in length of postoperative stay between both treatment groups mentioned by the studies separately [6, 8, 13]. Hall et al. [7] again showed median values and was therefore excluded. In our meta-analysis we also found no significant difference in postoperative hospital stay (mean difference = 2.41 h, 95% CI −6.10–1.28 h) (Fig. 5)*.*

## Discussion

There are still contradictory results in the literature with regard to the benefits and disadvantages of LP compared to the open procedure to treat infants with HPS. In our systematic review of high-level evidence, LP was found to be superior to OP with respect to a shorter time to full feed but not regarding (major) postoperative complications or length of hospital stay. Despite this small benefit, LP can be acknowledged as the standard of care only if the major postoperative complication rate is substantially reduced.

In this area of rapidly evolving technology, minimally invasive procedures are still a topic of interest in medicine. In pediatric surgery the number of minimally invasive procedures performed is still rising as more institutes in several countries are encouraged to do so. However, in the world of adult minimally invasive surgery, quite a few complications have occurred leading to criticism in the national health-care inspectorate [23]. More thorough research is therefore necessary to find out whether minimally invasive procedures are merely another technological improvement or a real clinical step forward. In pediatric surgery, surgical changes in the treatment of hypertrophic pyloromyotomy are a change from the classical RUQ incision toward the supraumbilical approach and, since 1991, the laparoscopic procedure as introduced by Alain et al. [4].

Recently, a systematic review and meta-analysis was published by Sola et al. [12] who compared complication rates and outcomes of laparoscopic and OP in infants with HPS. However, in our opinion, there are some shortcomings in their results. First, six prospective studies [5–8, 11, 13] were included, of which four were RCTs [6–8, 13]. Although, the studies by Fujimoto et al. [5] and Scorpio et al. [11] were prospective studies, they were not randomized and are therefore at higher risk of bias. However, these studies did not alter the results of the comparison of total complications in the OP and LP groups. Second, the study by Hall et al. [7] was included, although it used median results, and the other study [12] used mean values. This is a source of bias that weakens the results. Besides, one study reported was a prospective cohort study [11], which is statistically not comparable with RCTs in a systematic review. Again, there is a risk of potential bias. Furthermore, to our knowledge there is no randomized clinical trial or systematic review that described (major) complications as a primary outcome, which should be appreciated together with the possible positive outcomes.

It is important to note that every hospital has different standardized protocols for a feeding regimen, which makes an objective comparison difficult. A postoperative feeding schedule was started 6 h after recovery from anesthesia in the study of Hall et al. [7], while in the study of Leclair et al. [8], the feeding regimen was initiated 18 h after the operation. St. Peter et al. [13] maintained a feeding schedule in which feedings were started 2 h postoperatively. A breast-fed infant started ad libitum feeding 6 h after the pyloromyotomy in the study by Greason et al. [6].

The four studies selected for our review are RCTs, but they differ in the number of patients treated (Hall [7], 180; Leclair [8], 102; St. Peter [13], 200; and Greason [6], 20). In any procedure that is introduced into daily surgical care, new problems can occur during and after surgery that were not recognized or foreseen by the surgeon and his team. This learning curve in the four RCTs plays an underestimated role in major complications seen in the laparoscopic group, which may also have influenced secondary outcomes such as postoperative hospital stay. In our opinion RCT that compares two procedures, the surgeon(s) should be beyond the learning curve(s) of both operations in order to make a valid comparison. More learning curves should be defined for minimally invasive procedures [24] to make better studies possible to answer whether minimally invasive surgery is an alternative to open surgery or is the gold standard. This is not only mandatory for clinical reasons, but also when training new surgeons in these technically demanding procedures.

We know that for adult patients minimally invasive surgery has some benefits over open surgery by means of better cosmesis, body image, and postoperative complications [25–27]. Cosmesis and body image may be important to the parents of children with HPS, and later in life they may be important to our pediatric patients themselves.

In this systematic review we summarized all RCTs available in the pediatric surgical literature, focusing mainly on major complications that need surgical reintervention in patients with HPS. However, our results show no clear benefit of the laparoscopic procedure over the open operation. Time to full feed was found to be significantly shorter in the laparoscopic group, but this is measured in hours and therefore seems barely clinically significant. If the surgeon is able to perform both procedures, it is at the discretion of the surgeon or center to make a well-founded decision between the two options.
